# Associations of + 138 Ins/del A and + 5665 G/T polymorphisms of endothelin-1 gene with hypertension in Burmese people in Magway, Myanmar

**DOI:** 10.1186/s40885-022-00201-w

**Published:** 2022-07-15

**Authors:** Win Min Oo, Kyaw Thiha, Myat Mon Khine

**Affiliations:** Department of Biochemistry, University of Medicine, Magway, Myanmar

**Keywords:** Endothelin-1, Genetic polymorphism, Genotypes, Hypertension

## Abstract

**Background:**

Hypertension is one of the major public health problems worldwide, and is one of the recognized causes of premature deaths every year in the world. The purpose of this study was to investigate the associations between the + 138 insertion/deletion of adenine (Ins/del A) and + 5665 guanine-to-thymine (G/T) polymorphisms of the endothelin-1 gene and hypertension in the residents of Magway Township, Myanmar.

**Methods:**

This study was a cross-sectional comparative study including 60 hypertensive patients and 60 control subjects in Magway Township, Myanmar. The inclusion criterion for hypertension was blood pressure ≥ 140/90 mmHg or previous diagnosis by a physician as hypertension and/or taking antihypertensive drugs. The control group had blood pressure < 140/90 mmHg and no previous diagnosis of hypertension. The genotyping was done by polymerase chain reaction and restriction fragment length polymorphism method.

**Results:**

In this study, the genotype distribution of the + 138 Ins/del A variant was significantly different between hypertensive patients and the control group, especially in the 3A4A genotype (odds ratio [OR], 2.451; 95% confidence interval [CI], 1.138–5.280; *P* = 0.022). Adenine insertion genotypes (3A4A and 4A4A) were significantly associated with hypertension in the dominant model (OR, 2.494; 95% CI, 1.179–5.276; *P* = 0.017). In addition, there was a significant association between the 4A allele and hypertension (OR, 1.771; 95% CI, 1.026–3.056; *P* = 0.040). The genotype and allelic distributions of the + 5665 G/T polymorphism were not significantly different between the hypertensive patients and the control group (*P* > 0.05). In this study, there was no significant association between the genotype and allele frequency, and hypertension (*P* > 0.05). The linkage disequilibrium was weak between the + 138 Ins/del A and + 5665 G/T loci (D’ = 0.108, r^2^ = 0.009).

**Conclusions:**

This study provides evidence that the + 138 Ins/del A rather than + 5665 G/T polymorphism is associated with hypertension in Burmese people.

## Background

Hypertension is one of the most important public health problems in the world. The World Health Organization reported that high blood pressure causes premature deaths of 7.6 million people worldwide each year (13.5% of the global population) [1]. In 2010, the global prevalence of hypertension was estimated to be 31.1% (1.39 billion) [2]. The genetics of hypertension is complex, and many genes interact with various environmental factors. Butler stated that 30–50% of blood pressure variability depends on heredity [3]. In recent years, the polymorphisms of several candidate genes have been studied in hypertensive patients.

Endothelin-1 (EDN1) is a potent vasoconstrictor produced by the vascular endothelial cells [4, 5]. According to the available single nucleotide polymorphism (SNP) database, the *EDN1* gene (gene ID 1906, chromosome 6) contains 252 SNPs. Amongst these polymorphisms, two variants of the *EDN1* gene may play a role in the development of hypertension [6].

The insertion or deletion of an adenine (A) nucleotide at the position + 138 in 5’ untranslated region (rs1800997) is associated with blood pressure variation [7]. The 3A4A and 4A4A genotypes (mutant genotypes) were associated with hypertension in the UK (*P* < 0.05) [8]. However, the + 138 insertion/deletion of adenine (Ins/del A) polymorphism was not significantly associated with hypertension in Chinese and Czechs [9–11]. Another polymorphism, the guanine-thymine transversion at nucleotide + 5665 in exon 5 (rs5370), changes lysine to asparagine at codon 198 (also called Lys198Asn polymorphism) [12]. Compared with the GG variant (wild genotype), the TT genotype (mutant genotype) was associated with hypertension in Kazakhstan and Japan [13, 14]. However, the + 5665 guanine-to-thymine (G/T) variation was not associated with hypertension in Chinese people [9].

In the view of location and ethnicity, we assume that it would be prudent to investigate the association between + 138 Ins/del A and + 5665 G/T polymorphisms in *EDN1* gene and hypertension in Burmese people living in Magway Township, Myanmar. To this goal, we used polymerase chain reaction and restriction fragment length polymorphism (PCR-RFLP) method to genotype these polymorphisms in the study population.

## Methods

The study was conducted after approval by the Research Ethics Committee of University of Medicine, Magway. The registration number of the approval was 3(1)/UMMG-ERC/2019 on February 1, 2019. Written informed consent was obtained from all participants in the study. Participants had the right to leave the study at any time without interrupting standard medical care.

The study population included hypertensive patients who were treated in the outpatient departments of Magway Teaching Hospital and Magway Regional Hospital in Myanmar. The control subjects were selected from the staff of the University of Medicine, Magway in Myanmar. Their blood pressure was less than 140/90 mmHg and there was no diagnosis of hypertension. The age of both groups was between 35 and 70 years. We excluded known cases of secondary hypertension (based on the physician’s previous clinical and laboratory diagnoses), pregnant women, and alcoholics from the study.

The required sample size was estimated by using the following formula [15].

$$ n={\frac{\left(M+1\right)\kern1em \left[\frac{z_{1-\frac{\alpha }{2}}\left(1+\psi \right)+2{z}_{1-\beta}\sqrt{\psi }}{\psi -1}\right]}{2 Mk\left(\psi +1\right){\overline{\pi}}_p\left(1-{\overline{\pi}}_p\right)}}^2 $$

where n = sample size, *ψ* = odds ratio, M = control: case = 1, ${\overline{\pi}}_p$= proportion of mutant genotypes in hypertensive patients, ${z}_{1-\frac{\alpha }{2}}$ = 1.96 (95% confidence interval), *z*_1 − *β*_ = 1.845 (80% of power of the test), $\mathrm{k}=1/\left[1+\left(\uppsi -1\right){\overline{\pi}}_p\right]$. According to previous studies, ${\overline{\pi}}_p$ was 0.208 and *ψ* was 3.485 for +138 Ins/del A polymorphism [16]. For +5665 G/T polymorphism, ${\overline{\pi}}_p$ was 0.15 and *ψ* was 24.728 [13]. Minimal sample size required was round up to 50. If the dropout rate is assumed to be 20%, a sample size of 60 participants is required for each group.

After a thorough explanation of the study, written informed consent was obtained from all participants. A brief medical history was recorded and the participants’ blood pressure was measured. Then, 3 mL of venous blood was drawn from the participants for genotyping.

Genomic DNA was extracted by using Norgen Blood DNA isolation kit, catalog number 46,380, 46,300 (Norgen Biotek, Thorold, ON, Canada) according to the manufacturer’s instruction. Genotyping was performed by PCR-RFLP method, ethidium bromide staining, and agarose gel electrophoresis, and then the DNA fragments were observed under a UV transilluminator. Fragments containing two loci were amplified using specific primers described in the previous papers [10, 13].

The + 138 Ins/del polymorphism was identified by digesting the PCR product with *Dra I* restriction enzyme, resulting in 3A3A (210-bp), 3A4A (210- and 187-bp), and 4A4A (187-bp). The + 5665 G/T polymorphism was identified by digesting the PCR product with *Nhe I* restriction enzyme to produce GG (93-bp), GT (93- and 116-bp), and TT (116-bp).

### Statistical analysis

Data was collected by using proforma, cleansed and recorded in master sheet. The data was analyzed by using R software version 4.0.0. Descriptive statistics of numerical variables were expressed as frequency tables. Categorical variables were expressed as a percentage. Multi-model analysis was performed to determine the allele frequency and genotype frequency in the dominant and recessive models. Minor allelic frequencies (MAF) were documented for the whole study population as well as for each group. The odds ratios (OR) with 95% confidence intervals (95% CI) were calculated to test the associations between the genetic variations and hypertension [17]. A P-value less than 0.05 is considered statistically significant. As the measures of linkage disequilibrium (LD) between the two loci, standardized coefficient of LD (D’) and the degree of association (r^2^) were determined using Haploview [18].

### Operational definitions

#### Control group

The control group included individuals with no history of hypertension and systolic blood pressure (SBP) < 140 mmHg and diastolic blood pressure (DBP) < 90 mmHg at two readings with a 5-minute interval between them.

#### Hypertensive patients

Hypertensive patients are individuals having SBP ≥ 140 mmHg and DBP ≥ 90 mmHg at two readings with a 5-minute interval between them or previously diagnosed with hypertension by physicians regardless of any blood pressure.

## Results

The general characteristics of the subjects in this study is shown in Table 1. Of the 60 hypertensive patients, the mean age was 49.77 ± 12.53 years. Thirty-seven subjects were male, and the rest were female patients. Out of a total of 60 hypertensive patients, only 39 participants had a positive family history of hypertension. The mean blood pressure was 152/94 ± 21/13 mmHg. In the control group consisting of 39 male and 21 female subjects, the average age was 47.03 ± 13.15 years. Of the 60 control subjects, 11 had a family with hypertension. The mean blood pressure in the control group was 120/78 ± 12/8 mmHg.

**Table 1:** General characteristics of hypertensive patients and control group

General characteristic	Hypertensive patient (*n* = 60)	Control group (*n* = 60)
Age (yr), mean ± SD	49.77 ± 12.53	47.03 ± 13.15
Sex		
Male	37	39
Female	23	21
Family history of hypertension		
Present	39	11
Absent	21	49
SBP (mmHg), mean ± SD	152 ± 21	120 ± 12
DBP (mmHg), mean ± SD	94 ± 13	78 ± 8

*SD* standard deviation, *SBP* systolic blood pressure, *DBP* diastolic blood pressure

Table 2 shows the genotype and allele frequency of the + 138 Ins/del A polymorphism in the study population, as well as multi-model analysis to test the association with hypertension. Obviously, the heterozygous 3A4A genotype was more common (61.67%) in hypertensive patients, while the homozygous 3A3A genotype was more common (51.67%) in the control group. The lower frequency genotypes in the two groups were all homozygous 4A4A (8.33% in the hypertensive patients and 5% in the control group). In the two groups, the major allele was the 3 A allele, and the minor allele was the 4 A allele. The MAF in the study population was 0.33 out of 240 alleles (0.39 in hypertensive patients and 0.27 in the control group).

**Table 2:** Associations of genetic and allelic frequencies of + 138 Ins/del A polymorphism with hypertension

Model		Hypertensive patient (*n* = 60)	Control group (*n* = 60)	OR	95% CI	P-value
Genotypic model	3A3A	18 (30.00)	31 (51.67)	Reference	-	-
	3A4A	37 (61.67)	26 (43.33)	2.451	1.138–5.280	0.022
	4A4A	5 (8.33)	3 (5.00)	2.870	0.612–13.453	0.182
Dominant model	3A3A	18 (30.00)	31 (51.67)	2.494	1.179–5.276	0.017
	3A4A + 4A4A	42 (70.00)	29 (48.33)			
Recessive model	3A3A + 3A4A	55 (91.67)	57 (95.00)	1.727	0.394–7.577	0.478
	4A4A	5 (8.33)	3 (5.00)			
Allelic model	3 A	73 (60.83)	88 (73.33)	1.771	1.026–3.056	0.040
	4 A	47 (39.17)	32 (26.67)			

Data are presented as number (%) unless otherwise specified

*Ins/del A* insertion/deletion of adenine, *OR* odds ratio, *CI* confidence interval

There was a significant association between the 3A4A genotype and hypertension (OR, 2.451; 95% CI, 1.138–5.280; *P* = 0.022). There was a positive association between 4A4A genotype and hypertension (OR, 2.870; 95% CI, 0.612–13.453) but it was not statistically significant (*P* = 0.182). This finding may be due to the small frequency observed in this study and the relatively small sample size, as evidenced by the wide confidence interval. Under the dominant model, the OR of the dominant genotype of the hypertensive patients to the control group was 2.494 (95% CI, 1.179–5.276; *P* = 0.017). However, in the recessive model, there was no significant association between the + 138 Ins/del A variant and hypertension (OR, 1.727; 95% CI, 0.394–7.577; *P* = 0.478). Hypertension was significantly associated with the 4 A allele (OR, 1.771; 95% CI, 1.026–3.056; *P* = 0.040).

Table 3 shows the genotype and allele frequency of the + 5665 G/T polymorphism in the study population. In the two groups, heterozygous GT genotype was the most common (45% in the hypertensive patients and 50% in the control subjects), while homozygous TT genotypes were the least common (18.33% in the hypertensive group and 11.67% in the control). In the two groups, the major allele is the G allele and the minor allele is the T allele. The MAF in the study population was 0.39 (0.41 in the hypertensive patients and 0.37 in the control subjects). In multi-model analyses, there was no significant association between the + 5665 G/T polymorphism and hypertension (*P* > 0.05).

**Table 3:** Associations of genetic and allelic frequencies of + 5665 G/T polymorphism with hypertension

Model		Hypertensive patient (*n* = 60)	Control group (*n* = 60)	OR	95% CI	P-value
Genotypic model	GG	22 (36.67)	23 (38.33)	Reference	-	-
	GT	27 (45.00)	30 (50.00)	0.941	0.430–2.057	0.999
	TT	11 (18.33)	7 (11.67)	1.643	0.540–5.002	0.389
Dominant model	GG	22 (36.67)	23 (38.33)	1.074	0.513–2.249	0.861
	GT + TT	38 (63.33)	37 (61.67)			
Recessive model	GG + GT	49 (81.67)	53 (88.33)	1.700	0.610–4.733	0.315
	TT	11 (18.33)	7 (11.67)			
Allelic model	G	71 (59.17)	76 (63.33)	1.192	0.709–2.005	0.518
	T	49 (40.83)	44 (36.67)			

Data are presented as number (%) unless otherwise specified

*G/T* guanine-to-thymine, *OR* odds ratio, *CI* confidence interval

Table 4 shows the measurement of LD between the + 138 Ins/del A and + 5665 G/T loci in *EDN1* gene in the study population. The LD between the two loci was weak (D’ = 0.108, r^2^ = 0.009).

**Table 4:** Linkage disequilibrium between + 138 Ins/del A and + 5665 G/T polymorphisms of *EDN1* gene

Determinant	Total
D’	0.108
r^2^	0.009

*Ins/del A* insertion/deletion of adenine, *G/T* guanine-to-thymine

## Discussion

The + 138 Ins/del A polymorphism is located at nucleotide position + 138 of 5’ untranslated region of *EDN1* gene in chromosome 6 (chr6:12290496–12290499, GRCh38.p12) and its reference SNP number is rs1800997. Because it is located in the non-coding region of the *EDN1* gene, the amino acid sequence of the endothelin precursor is not affected [19].

A significant association was observed between the + 138 Ins/del A polymorphism and hypertension in this study (*P* < 0.05) (Table 2). The results of this study are consistent with a previous study conducted in the UK, which revealed a significant association between the insertion genotype and hypertension (χ^2^ = 6.202, *P* = 0.045) [8]. However, some other previous studies have produced conflicting results. In another study conducted in the UK, the + 138 Ins/del A polymorphism was not associated with hypertension (*P* > 0.05) [20]. Correspondingly, there was no association between + 138 Ins/del A polymorphism and hypertension in the Czech, Chinese and Americans in previous reports [9–11, 21]. Hence, it can be proposed that interethnic differences may influence the association of the + 138 Ins/del A polymorphism with hypertension.

The mechanism underlying the association between + 138 Ins/del A polymorphism and hypertension in this study may be the influence of gene expression by the + 138 Ins/del A polymorphism. Popowski et al. [7] revealed the effect of the + 138 Ins/del A polymorphism on mRNA and protein formation in experimental studies. The 4A4A genotype was significantly associated with elevated mRNA level compared to the 3A3A genotypes (*P* < 0.001). EDN1 protein expression was also higher among the 4A4A genotypes (*P* < 0.001). These findings may be due to increased mRNA stability and increased gene expression, because the half-life of mRNA in the 4A4A genotype was long as compared with other genotypes (35.4 ± 7.9 vs. 19.9 ± 4.5 min) [7]. Therefore, the current study agrees with the mechanism explored by Popowski et al. [7].

The + 5665 G/T polymorphism is located at nucleotide position + 5665 of exon of *EDN1* gene on chromosome 6 (chr6:12296022, GRCh38.p12), and its reference SNP number is rs5370 [22]. The transversion of guanine-to-thymine changes lysine to asparagine at amino acid 198 of EDN1 protein. The locus is not in the regulatory region of the *EDN1* gene, but in the region encoding prepro-endothelin-1 [23].

In the present study, there was no significant association between the + 5665 G/T polymorphisms and hypertension (*P* > 0.05) (Table 3). In the multi-model analysis, no significant association was observed between the + 5665 G/T gene variation and hypertension (*P* > 0.05). Correspondingly, the alleles were not significantly associated with hypertension (*P* > 0.05).

These findings were consistent with a previous study on Chinese people conducted by Fang et al. [9]. In a total of 423 hypertensive subjects and 114 healthy control subjects, there was no significant association between genotype frequency and hypertension (OR, 0.84; 95% CI, 0.51–1.37; *P* = 0.541). In addition, there was no significant association between allele frequency and hypertension (OR, 1.28; 95% CI, 0.81–2.01; *P* = 0.323) [9]. In contrast, in the study of Dzholdasbekova and Gaipov [13] consisting of 120 Kazakh hypertensive patients and 80 controls, the T allele and the TT genotype were significantly associated with hypertension (χ^2^ = 13.81, *P* = 0.001 in genotypic model; χ^2^ = 7.27, *P* = 0.007 in allelic model).

This study did not reveal any association between the + 5665 G/T genotype and hypertension because there may be inter-ethnic differences that affect the association between genetic polymorphism and hypertension. Another possible explanation of the + 5665 G/T polymorphism affecting the blood pressure is that genotype-phenotype association may be influenced by environmental factors such as obesity, fitness level, physical activity and socioeconomic status which were not evaluated in the present study [12, 14, 24–27]. The sample size required to evaluate gene-environment interactions is much larger than the sample size required to determine genetic or environmental factors alone. Although the current study did not investigate the effect of environmental factors on genotype-phenotype association, it showed no association between the + 5665 G/T polymorphism itself and hypertension and this result can provide information for future research that will consider the interaction with the environmental factors.

In the present study, the D’ between the + 138 Ins/del A and + 5665 G/T polymorphisms was 0.108 and the r^2^ was 0.009 (Table 4). Hence, in this study, LD between these two loci was weak. In previous data, LD between the two loci was found to be weak in some populations including Gujarati Indian from Houston, Texas; Punjabi from Lahore, Pakistan; Indian Telugu from the UK; Kinh in Ho Chi Minh City, Vietnam; Chinese Dai in Xishuangbanna, China; Sri Lankan Tamil from the UK; and Bengali from the Bangladesh (r^2^ < 0.05, D’ < 0.05). Meanwhile, some other populations revealed strong LD between the two variants as in Europeans and Africans (r^2^ > 0.5, D’ > 0.5). These data are currently available by using the interactive LDpop tool online [28]. Based on the results of this study and previous reports, it is clear that the LD between the + 138 Ins/del A and + 5665 G/T polymorphisms shows a population-specific difference. There can be no chromosome-specific differences between these two loci because they are located on the same chromosome (chromosome 6) [29].

The limitations of this study were that the sample size was relatively small, the selection of control individuals was not strict with ambulatory blood pressure measurements, and environmental factors were not investigated. However, this study can prove that hypertension is significantly related to the + 138 Ins/del A polymorphism of *EDN1* gene, while the effect of the + 5665 G/T polymorphism is not significant on hypertension in Burmese people, Myanmar.

## Conclusions

This study revealed that the + 138 Ins/del A polymorphism of the *EDN1* gene is significantly associated with hypertension in Burmese living in Magway Township, Myanmar. Under the allelic and dominant models, mutant genotypes were associated with hypertension. On the other hand, the + 5665 G/T polymorphism has no effect on hypertension. In addition, the + 138 Ins/del A and + 5665 G/T polymorphisms of *EDN1* gene are not in strong LD. This research provides genetic information for the development of an advanced database of hypertension genetics, enabling biomedical scientists and clinicians to learn more about the contribution of genetics to hypertension.

## Data Availability

The datasets used and/or analyzed during the current study are available from the corresponding author on reasonable request.

## Abbreviations

A: Adenine
CI: Confidence interval
D’: Standardized coefficient of linkage disequilibrium
DBP: Diastolic blood pressure
EDN1: Endothelin-1
G: Guanine
G/T: Guanine-to-thymine
Ins/del A: Insertion/deletion of adenine
LD: Linkage disequilibrium
MAF: Minor allele frequency
OR: Odds ratio
PCR-RFLP: Polymerase chain reaction and restriction fragment length polymorphism
r^2^: Degree of association
SBP: Systolic blood pressure
SNP: Single nucleotide polymorphism
T: Thymine

## References

[1] World Health Organization. Global health risks: mortality and burden of disease attributable to selected major risks. 2009. https://apps.who.int/iris/handle/10665/44203. Assessed 12 Jan 2021.

[2] Mills KT, Stefanescu A, He J (2020). The global epidemiology of hypertension. Nat Rev Nephrol.

[3] Butler MG (2010). Genetics of hypertension: current status. J Med Liban.

[4] Inoue A, Yanagisawa M, Kimura S, Kasuya Y, Miyauchi T, Goto K (1989). The human endothelin family: three structurally and pharmacologically distinct isopeptides predicted by three separate genes. Proc Natl Acad Sci U S A.

[5] Kohan DE (2010). Endothelin, hypertension and chronic kidney disease: new insights. Curr Opin Nephrol Hypertens.

[6] Ahmed M, Rghigh A (2016). Polymorphism in endothelin-1 gene: an overview. Curr Clin Pharmacol.

[7] Popowski K, Sperker B, Kroemer HK, John U, Laule M, Stangl K (2003). Functional significance of a hereditary adenine insertion variant in the 5’-UTR of the endothelin-1 gene. Pharmacogenetics.

[8] Stevens PA, Brown MJ (1995). Genetic variability of the ET-1 and the ETA receptor genes in essential hypertension. J Cardiovasc Pharmacol.

[9] Fang Z, Li M, Ma Z, Tu G (2017). Association of endothelin-1 gene polymorphisms with essential hypertension in a Chinese population. Genet Mol Res.

[10] Fan XH, Wang H, Gao LG, Sun K, Zhou XL, Hui RT (2012). The association of an adenine insertion variant in the 5’UTR of the endothelin-1 gene with hypertension and orthostatic hypotension. Arch Med Sci.

[11] Vasku A, Tschöplova S, Muzik J, Soucek M, Vacha J (2002). Association of three polymorphisms in the gene coding for endothelin-1 with essential hypertension, overweight and smoking. Exp Clin Cardiol.

[12] Tiret L, Poirier O, Hallet V, McDonagh TA, Morrison C, McMurray JJ (1999). The Lys198Asn polymorphism in the endothelin-1 gene is associated with blood pressure in overweight people. Hypertension.

[13] Dzholdasbekova A, Gaipov A (2010). The association between polymophism of Lys198Asn of endothelin-1 gene and arterial hypertension risk in Kazakh people. Euro J Gen Med.

[14] Jin JJ, Nakura J, Wu Z, Yamamoto M, Abe M, Tabara Y (2003). Association of endothelin-1 gene variant with hypertension. Hypertension.

[15] Parker RA, Bregman DJ (1986). Sample size for individually matched case-control studies. Biometrics.

[16] Vadapalli S, Rani HS, Sastry B, Nallari P (2010). Endothelin-1 and endothelial nitric oxide polymorphisms in idiopathic pulmonary arterial hypertension. Int J Mol Epidemiol Genet.

[17] Lewis CM (2002). Genetic association studies: design, analysis and interpretation. Brief Bioinform.

[18] Barrett J, Fry B, Maller J, Daly M (2004). Haploview: analysis and visualization of LD and haplotype maps. Bioinformatics.

[19] National Center for Biotechnology Information. dbSNP short genetic variations. Reference SNP (rs) report: rs1800997. 2020. https://www.ncbi.nlm.nih.gov/snp/rs1800997. Accessed 20 Dec 2020.

[20] Brown MJ, Sharma P, Stevens PA (2000). Association between diastolic blood pressure and variants of the endothelin-1 and endothelin-2 genes. J Cardiovasc Pharmacol.

[21] Lassen O, Herrera J, Dotto (2016). Plasmatic biochemical variables associated with polymorphisms in the endothelin-1 and endothelin-1 receptor a genes in hypertensive patients: pilot study. Br J Med Med Res.

[22] National Center for Biotechnology Information. dbSNP short genetic variations. Reference SNP (rs) report: rs5370. 2020. https://www.ncbi.nlm.nih.gov/snp/rs5370. Accessed 20 Dec 2020.

[23] Colombo MG, Ciofini E, Paradossi U, Bevilacqua S, Biagini A (2006). ET-1 Lys198Asn and ET(A) receptor H323H polymorphisms in heart failure: a case-control study. Cardiology.

[24] Asai T, Ohkubo T, Katsuya T, Higaki J, Fu Y, Fukuda M (2001). Endothelin-1 gene variant associates with blood pressure in obese Japanese subjects: the Ohasama Study. Hypertension.

[15] Treiber FA, Barbeau P, Harshfield G, Kang HS, Pollock DM, Pollock JS (2003). Endothelin-1 gene Lys198Asn polymorphism and blood pressure reactivity. Hypertension.

[26] Rankinen T, Church T, Rice T, Markward N, Leon AS, Rao DC (2007). Effect of endothelin 1 genotype on blood pressure is dependent on physical activity or fitness levels. Hypertension.

[27] Tanaka C, Kamide K, Takiuchi S, Kawano Y, Miyata T (2004). Evaluation of the Lys198Asn and – 134delA genetic polymorphisms of the endothelin-1 gene. Hypertens Res.

[28] Alexander TA, Machiela MJ (2020). LDpop: an interactive online tool to calculate and visualize geographic LD patterns. BMC Bioinformatics.

[29] Zavattari P, Deidda E, Whalen M, Lampis R, Mulargia A, Loddo M (2000). Major factors influencing linkage disequilibrium by analysis of different chromosome regions in distinct populations: demography, chromosome recombination frequency and selection. Hum Mol Genet.

